# Sustainable financing mechanisms for strengthening mental health systems in Nigeria

**DOI:** 10.1186/s13033-019-0293-8

**Published:** 2019-05-31

**Authors:** Jibril Abdulmalik, Saheed Olayiwola, Sumaiyah Docrat, Crick Lund, Dan Chisholm, Oye Gureje

**Affiliations:** 1 Department of Psychiatry, College of Medicine, University of Ibadan, Ibadan, Nigeria; 2 Department of Economics, Federal University of Technology, Akure, Nigeria; 3 Alan J Flisher Centre for Public Mental Health, Department of Psychiatry and Mental Health, University of Cape Town, Cape Town, South Africa; 4 Centre for Global Mental Health, Health Service and Population Research Department, Institute of Psychiatry, Psychology and Neuroscience, King’s College London, London, UK; 5 Department of Mental Health and Substance Abuse, World Health Organization, Geneva, Switzerland; 6 World Health Organization Collaborating Centre Collaborating Centre for Research and Training in Mental Health, Neurosciences and Substance Abuse, Department of Psychiatry, College of Medicine, University of Ibadan, Ibadan, Nigeria

**Keywords:** Mental health financing, Nigeria, Mental health systems, Low income countries, Global mental health

## Abstract

**Background and aims:**

Current coverage of mental health care in low- and middle-income countries is limited, not only in terms of access to services but also in terms of financial protection of persons in need of care and treatment. This is especially pertinent considering the established relationship between mental illness and poverty and the need to ensure the financial risk protection of persons with mental disorders and their families as part of country’s efforts to attain universal health coverage. This study set out to review the health and socio-economic contexts of Nigeria as well as to generate strategies for sustainable mental health financing that will be feasible, within the specific context of the country.

**Methods:**

A multi-methods approach was developed and applied, consisting of three steps: a situational analysis of Nigeria’s health system, macro-fiscal economic profile, and socio-political status, including a strengths, weaknesses, opportunities and threats (SWOT) analysis of the Nigerian socio-economic, general and mental health context; key informant interviews with 12 expert stakeholders drawn from state and non-state actors in the health and financial sectors; and a policy analysis of sustainable financing options.

**Results:**

Key challenges identified were: poor funding; reduced access to care, resulting in a huge treatment gap; and out of pocket payment for services—leading to impoverishment. Comprehensive coverage of mental health conditions within the ongoing health insurance reforms was identified as a key strategy for moving towards sustainable mental health financing in Nigeria. Other identified strategies include enhanced integration of mental health into primary care; incorporation of mental health into other strategic and currently funded programmes; adoption of performance-based financing measures; and renewed engagement with stakeholders, including external donor institutions.

**Conclusions:**

A suite of feasible and actionable measures can be implemented to increase mental health service financing, reduce health-related financial burden on households, increase help-seeking and access to quality mental health care and, ultimately, reduce the large treatment and financing gap for mental disorders in Nigeria.

## Introduction

Effective and optimally functional health systems should deliver high-quality services to all persons, whenever and wherever they need such services [1]. The goal of attaining universal health coverage (UHC) is anchored on achieving improvements in health status, equitable access to health care, fair financing, service quality and human rights protection. Successful attainment of UHC is a unifying goal for health system strengthening, as well as an explicit target for the sustainable development goals (SDGs) which are anchored on the principle of not leaving anyone behind [2].

The concept of UHC encompasses two major areas: service coverage and financial coverage. With respect to service coverage for mental disorders, the treatment gap for low- and middle-income countries (LMICs) is between 76 and 85%, with the figures for Nigeria indicating that only 1 out of every 5 persons with a mental disorder is able to access any care [3]. However, this huge treatment gap is not entirely due to the unavailability of services alone, but is also partly due to a lack of financial means to pay for such services (poor financial coverage). Thus, persons or households with lower incomes, greater vulnerability and longer-term health care needs are particularly affected. This is especially true in Nigeria and other LMICs where out-of-pocket (OOP) payments remain the most common mode of procuring health care services. The OOP in Nigeria as at 2015, was about 72% [4].

Thus, LMICs suffer from the dual challenges of reduced service coverage as well as limited financial protection for mental, neurological, and substance use (MNS) disorders; in addition to pronounced adverse economic consequences for households [5]. The relationship between mental illness and poverty has been extensively evaluated in high income countries, and its impact on quality of life, increased economic burden of care, and reduced productivity, all of which ultimately reinforces poverty have been documented [6, 7]. The financial impact is even more striking, because the onset of the majority of mental illnesses occurs in early adulthood, a period when individuals should be at the peak of their economic productivity [8]. The association between mental illness and lost productivity, as well as eventual risk of poverty has also been demonstrated in the sub-Saharan African countries of South Africa, Nigeria and Ghana [9–11].

The high and potentially catastrophic cost to households of procuring the health care services they need is a fundamental concern underlying the drive toward UHC. Direct OOP payments penalize those least able to afford care—and directly lead to impoverishment. Indeed, the World Health Organization estimates that 100 million people suffered catastrophic health care expenditure and were directly pushed below the poverty line, subsisting on less than 2 US dollars per day, in 2010 [1].

The current study is a component of the project, *Emerging mental health systems in low and middle*-*income countries (Emerald)*, which aims to improve mental health outcomes in LMICs by identifying barriers within health systems and generating solutions [12]. It specifically addresses 3 key objectives: (a) adequate, fair and sustainable resourcing (health system inputs); (b) integrated physical and mental health service provision including capacity building (health system processes); and (c) improved coverage of care and goal attainment (health system outputs). The Emerald project was implemented in the 6 LMICs of Ethiopia, India, Nepal, Nigeria, South Africa and Uganda.

This paper reports on findings from the first Emerald objective. Successful mental health system strengthening that delivers UHC will be impossible without guaranteeing financing support in a sustainable manner. The Emerald project has approached this challenge by addressing a number of related health systems financing issues, including the sufficiency of resources for mental health, fairness in financial contributions to the costs of care, and the financial and economic impact of improved access to services. The estimates of the resource needs and the costs and health impacts of scaled-up mental health service delivery have been generated in each of the six participating Emerald countries [13]. Although previous research in Nigeria has documented the prevalence of mental illness and estimated the cost of scaling up packages of care, little is known about the dimensions of UHC pertaining to mental health and optimal financing strategies to ensure financial risk protection for people living with mental illness. This paper provides a situational analysis of where Nigeria lies, both with respect to the key dimensions of UHC (financial risk protection and access to services) and the broader health system characteristics, constraints, determinants and capacities, including the macroeconomic and fiscal environment. It outlines the main findings on mental health financing and equity, including exploration of potential strategies for increased financial protection and recommend strategies for moving towards universal health coverage for persons with mental disorders, taking current and projected needs, constraints and opportunities in the Nigeria into consideration.

## Methods

### Study design

The study utilized a sustainable financing framework that was developed by the Emerald project. This involved a streamlined, stepped approach to informing and evaluating financing needs in the area of mental health. The key dimensions of this framework included the following steps: (i) an assessment of the economic consequences of mental disorders; (ii) a critical appraisal of current and proposed governance, service delivery and financial protection arrangements for the treatment and prevention of mental disorders; (iii) an assessment of the current and projected macro-fiscal situation; (iv) an evaluation of projected resource needs for mental disorders; and (v) identification and selection of appropriate financing mechanisms. Some of these steps (economic consequences as well as projected resource needs for mental disorders) had been concluded and published elsewhere [13]; while this study focused on providing an overview of the governance structures, health, economic and political contexts of Nigeria, via a situational analysis. Subsequently, key informant interviews were conducted to identify and select financing mechanisms that are country-specific and feasible for the attainment of sustainable mental health financing in Nigeria.

### Data collection

Data collection was conducted in two phases: (a). situational analysis and (b) key informant interviews.

A. Situational analysis: This was performed to provide an overview of the overall health, mental health, economic—including health financing, and political context of Nigeria. A desk review of available government documents on health and mental health policies, legislation and services; as well as economic reports were obtained from government offices (where they were not available in the public domain) and reviewed. Furthermore, published as well as grey literature on these domains were also retrieved and reviewed. Global databases with country specific profiles and reports such as the World Bank and the World Health Organization (WHO) were also utilized as sources of data to enrich this overview. Deriving from this situational analysis, a strength, weakness, opportunities and threats (SWOT) analysis for sustainable mental health financing in Nigeria was mapped out.
B. Key Informant Interviews: Relevant stakeholders were identified and approached for key informant interviews (KII) at national and regional levels. Theses stakeholders were carefully selected state and non-state actors drawn from health, finance, other relevant government agencies, academia, development partners (WHO and the World Bank) and non-governmental organizations (NGOs) to ensure inclusiveness and wide representation. Purposive sampling was implemented to identify and select 12 stakeholders (8 national and 4 regional), that were approached for these interviews. Interviewees were all senior policymakers, provided informed consent and were interviewed face-to-face in English language, the selection of the respondents was guided by a need for inclusiveness, relevant expertise and the involvement of all government and non-governmental bodies with key influence and experience of the Nigerian health, financial and economic context.

A semi structured mental health financing diagnostic tool was utilized for the interviews, which covered three main themes: (a) perceived constraints to increased public health financing—including mental health (b) options for increased public health financing (and mental health), and (c) critical elements for improved public health (and mental health) financing. Sub themes covered under these broad domains included level of priority accorded to mental health; current health financing systems—including extent of mental health coverage, budgetary processes; impact of macro-economic issues; perceived challenges to increased financing for scaling up mental health services; suggested recommendations for the attainment of improved and sustainable financing for public health generally and for mental health services specifically; given their intimate knowledge of the Nigerian Context; and lastly, the key elements that should be in place for these recommendations to succeed. The interviews were audio-recorded and lasted for 45 min on the average.

### Ethics

Ethical approval was obtained from the University of Ibadan Health Research and Ethics Committee and the aims of the study were explained and written informed consent was obtained from all participants prior to the commencement of the interviews. The names and other personal identifiers of the participants were not required and the responses identified by means of codes to ensure confidentiality of the responses. Their permission was obtained for the audio recording with the assurance that the tapes will be securely locked and destroyed after transcription and data analysis. Furthermore, participants were re-assured that the questions were health system related rather than personal, and were therefore, unlikely to cause personal discomfort.

### Data analysis

The interview audio recordings were transcribed verbatim and analysed using a set of pre-determined a priori coding framework with the following high-level themes: challenges to increasing health (and specifically, mental health) financing; opportunities and strategies for improving public health (and mental health) financing; and key considerations that are essential for successful implementation. The data from the situational analysis, SWOT mapping as well as the KIIs were summarized to generate policy recommendations and strategies for sustainable mental health financing in Nigeria.

## Results

### Situational analysis

#### General health

Due to the persistently unacceptable health indices in the country over the years, the Federal Ministry of Health (FMOH) has embarked upon a rigorous health sector reform process, which has identified the improvement of access to quality health care as one of its seven major thrusts. It is also addressing the issue of equity as well as improving community awareness in the provision of health care delivery services in the country. But the major problems of brain drain and inadequate numbers of health professionals remain largely unsolved [14]. For example, the total number of physicians in 2007 was about 52,408 which translates to 3.7 per 10,000 population; while nurses and midwives were 219,407, which translates to a density of about 15.5 per 10,000 population. These ratios are well above the sub-Saharan averages of 1.5 doctors and 7.2 nurses per 10,000 population for the same year [15]. However, by 2017, only 39,912 doctors renewed their annual practising licence with the Medical and Dental Council of Nigeria, out of a total of 86,722 on their books [via telephone interview with an official of the Council]. This is equivalent to 2.2 per 10,000 population, and is due in significant part, to migration out of the country.

#### Mental health situation

The largest epidemiological survey from Nigeria estimates the lifetime prevalence of mental, neurological and substance use (MNS) disorders at 12.1%. This was part of the WHO World Mental Health Surveys conducted across all regions of the world. Results indicate that 5.8% had a 12-month prevalence of a mental disorder. The implication of these rates is that 21.8 million Nigerians are at risk of developing a mental disorder at some point in their lifetime; while 10.4 million Nigerians may be suffering from at least one mental disorder in any given year. Twenty-three percent (23%) had serious and disabling disorders, out of whom only 8% had received treatment in the preceding 12 months [16]. It should be noted that this data is nearly 13 years old, and no other nationally representative epidemiological studies have been conducted thereafter. However, estimates from the Global Burden of Disease (GBD) study, indicate that the point prevalence for MNS disorders may have doubled to 10.65%, or 20.8 million Nigerians with an MNS disorder; using country data generated for 2016 [17].

The existing legislation on mental health was derived from the British colonial laws of 1916, which were adopted and enacted in 1958 as the Lunacy Act, CAP 112, Laws of the Federation of Nigeria [18]. However, a revised National Mental Health Bill has been drafted and has been awaiting legislative action at the National Assembly for nearly a decade. The passage of the legislation may significantly improve financing, delivery of mental health services and the protection of the human rights of persons with mental disorders in Nigeria. There is cautious optimism that this legislation will be passed soon, as a result of concerted advocacy efforts and increasing public awareness and political will. This optimism is premised on a coalition that is led by the Association of Psychiatrists in Nigeria, with support from Corporate Organizations led by a Telecommunications giant (MTN), in partnership with some Legislators, that is now championing this cause. The first National Mental Health Policy was ratified in 1991 and was revised in 2013. The Mental Health Policy acknowledges the insufficient numbers of mental health professionals available in the country and recommends the integration of mental health into primary health care as a policy objective. Intersectoral collaboration and the creation of a Directorate for Mental Health at the Federal Ministry of Health are also key recommendations in the revised mental health policy. Aspects that have been implemented thus far include the appointment of a Mental Health Desk Officer at the National-level as well as Regional (State) levels, and; increasing the number of states that are involved in research-led projects to integrate mental health into primary and general medical care.

There is no specific budget line for mental health but in 2006 it was estimated that approximately 3.3% of the nation’s health budget goes towards funding stand-alone specialist neuropsychiatric facilities [19]. This proportion has remained fairly constant over time, using the same methods of estimation—the total amount allocated to mental health (calculated crudely as the budgetary allocation to all neuropsychiatric facilities) as a fraction of the total health budget), which amounted to about N10.2b ($28.7m at N355 per $1) in the 2017 budget [20]. Nigeria has very low numbers of mental health professionals for her rapidly growing population of nearly 200 million people. The World Mental Health Atlas of 2014 indicate that the psychiatrist to population ratio is about 1:1.6 million citizens. The figures for other mental health professionals are equally dismal, with 7:1 million for nurses; 1:5 million for psychologists, 2:5 million for social workers, and 1:10 million for occupational therapists [18]. This remains the most recent available source of information, as the World Mental Health Atlas of 2017 has minimal information in its country profile for Nigeria. Private mental health care service provision is limited to a few major cities in the country, and are often expensive, with limited scope and coverage. Roughly less than 5% of mental health professionals in the country work in the private sector, and therefore the private sector does not represent a significant source of competition or drain on the available numbers of mental health professionals in public-sector. This was estimated crudely, using the number of psychiatrists working in private practice (about 15), as a fraction of the total number of psychiatrists in the country (about 300).

The protracted insurgency by the Boko Haram in the north-east region of the country has resulted in devastating loss of lives, destruction, and internal displacement of nearly 4 million persons, and over 20,000 fatalities. The trauma has ranged from killings and displacements to kidnappings of young school girls, as well as attendant food shortages and reduced access to both physical and mental health services [21]. The response has been concerted with a mental health and psychosocial response intervention effort, championed by the Federal Government (FG), with support from the WHO, as well as non-governmental organizations such as the International Organization for Migration (IOM) and other international partners. Funds from these donor agencies currently finance the rebuilding efforts, with a focus on ‘building back better’. A key strategic partner has been the state governments, with technical support from the WHO, as exemplified by the production of a Mental Health Strategic Framework for Borno State; as well as the Federal Neuropsychiatric Hospital in Maiduguri, Borno State [22].

#### Health financing

The basic health care financing strategy options are general and earmarked taxes, social insurance, private insurance, community financing and out-of-pocket payments. The chosen financing strategy by any country determines how much money is available, who bears the financial burden and controls the funds and whether health expenditure inflation can be managed [23].

**Fig. 1:**
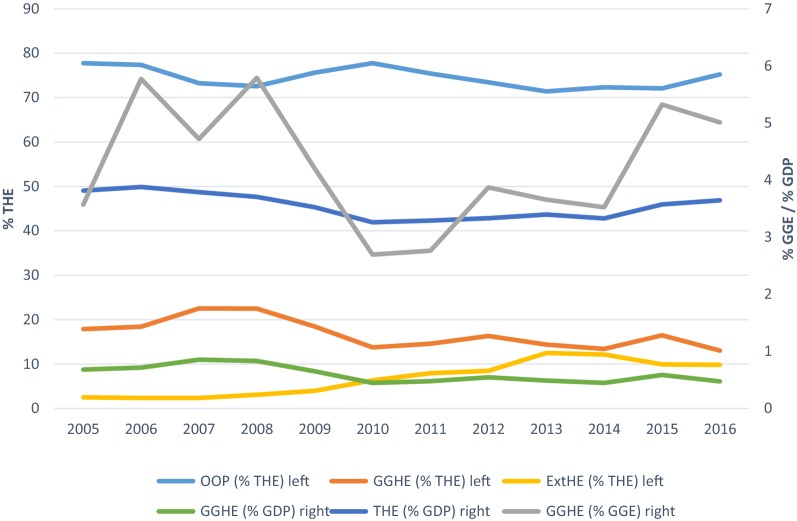
Health expenditure trends in Nigeria: 2005–2016. Source: Global Health Expenditure Database, World Health Organization [24]

The commonest modality of health care financing in Nigeria, remains out of pocket expenditure—accounting for over 70% of health care expenditure between 2005 and 2016 [24]. Over the same time period, current health expenditure as a percentage of Gross Domestic Product (GDP) was between 3.81 and 3.65%. Furthermore, federal government expenditure as percentage of current health expenditure ranged between 13 and 23% over the same period; while domestic general government health expenditure as a percentage of GDP hovered between 0.68 and 0.83% from 2005 to 2016. See Fig. 1.

#### Economy

**Fig. 2:**
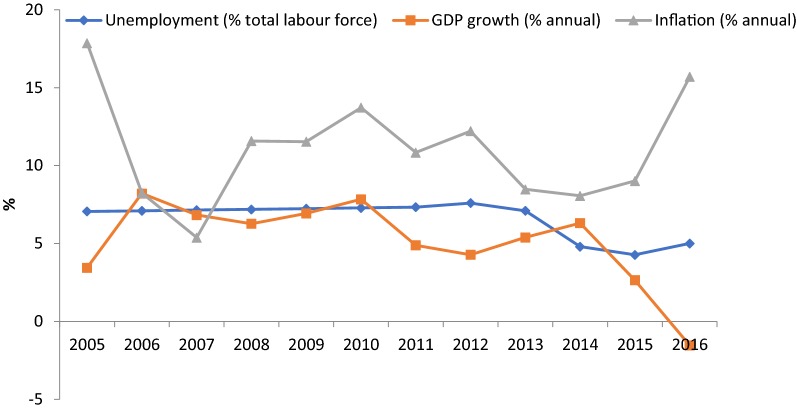
Macroeconomic situation in Nigeria: 2005–2016. Source: World development indicators [4]

Nigeria is heavily dependent on oil revenues, with oil and gas accounting for over 90% of Nigeria’s exports and more than 70% of consolidated budgetary revenue [25]. Nigeria faces a difficult short- and medium-term macroeconomic outlook, but has the opportunity to make major progress towards more diversified development and greater efficiency in public finance. Higher growth in Nigeria was expected gradually after the economic recession of 2015–2016. A shrinking resource envelope due to falling oil prices, prompted the FG to introduce significant cuts and adjustments to the 2014 and 2015 budgets with a particular focus on reducing capital expenditures. Resource allocations to priority social sectors such as education and health were protected in the 2015 and 2016 budgets. Health spending marginally improved from 5.0% in 2014 to 5.1% in 2016 [26]. Figure 2 further shows the macroeconomic situation in Nigeria. It shows high and fluctuating levels of inflation from 2005 to 2016 on the one hand; and comparatively high levels of inflation and unemployment, when compared to economic growth, which nosedived, as the nation plunged into a recession from 2015 to 2016.

**Fig. 3:**
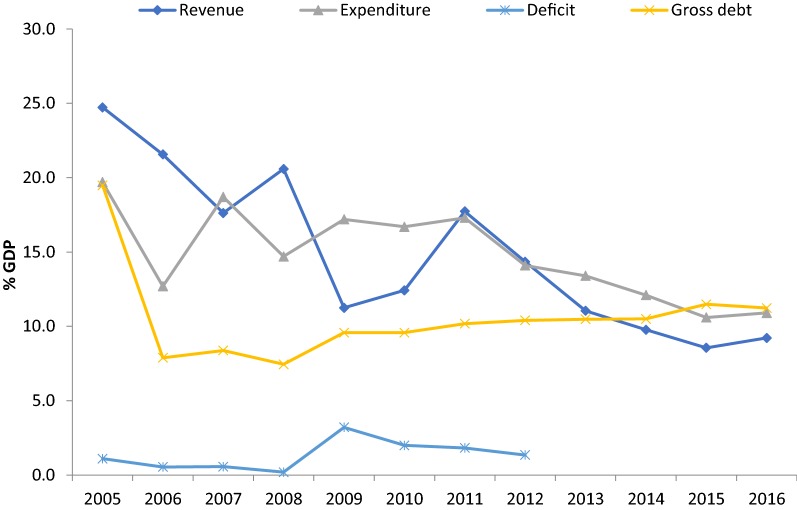
Fiscal indicators in Nigeria: 2005–2016. Source: World development indicators [4]

Furthermore, Nigeria recorded a government Debt to GDP ratio of 10% in 2016. Government Debt to GDP in Nigeria averaged 34.26% from 2000 until 2016, reaching an all-time high of 88% in 2001 and a record low of 10% in 2016. The future of the country’s external balance remains uncertain, though the depreciation of the naira and the imposition of capital controls appear to have been sufficient to restore general balance of payments equilibrium. Rising oil prices or increased capital inflows could also have a positive impact on the external balance [26]. Figure 3 shows the fiscal indicators in Nigeria, indicating that government expenditures have been consistently greater than revenue over the past decade.

Nigeria is one of the top five countries with the largest number of poor citizens, and is ranked third in the world, in a listing of where 7% of the world’s poor citizens live. In the Human Development Index (HDI) ranking for 2014, Nigeria is ranked 152 out of 187 countries. Poverty still remains one of the most critical challenges facing the country; and population growth rates have meant a steady increase in the number of poor citizens. Nearly a 100 million people live on less than a $1 (£0.63) a day, despite economic growth [25].

Politically, the country runs a federal system of government, with 36 states and a federal capital territory in Abuja. Health is on the recurrent legislative list in the constitution, which means that both the federal, state and local governments have shared responsibilities in this area. While corruption and insecurity have been the major threats to economic growth and political stability, government has in recent years placed a major focus on attending to these threats. These efforts lend credence to cautious optimism that there will be positive change—particularly with respect to the health sector in general, and mental health specifically.

### Opportunities for sustainable mental health financing

A critical SWOT analysis to highlight the current strengths, weaknesses, opportunities and threats for sustainable mental financing in Nigeria was performed in the light of the contextual overview of the country already presented. The SWOT analysis and possible opportunities for scaling-up sustainable financing for mental health in Nigeria are highlighted in Tables 1 and 2.

**Table 1:** Strengths and weaknesses of the Nigerian Context

Strengths	Weaknesses
*Health system factors*	*Health system factors*
Rigorous health sector reforms process Increasing State/regional awareness about Mental Health and the need to attend to it Health and social services are a priority for government	Inadequate access to formal health care services Reduced utilization due to pervasive stigma Inadequate numbers of mental health professionals
*Economic factors*	*Economic factors*
Likelihood of economic growth and recovery from recession More accountability due to government’s war against corruption Increasing Foreign Direct Investment due to the country’s political stability and increasing positive reputation internationally Health insurance schemes have commenced and efforts are being made to cover the vulnerable	Mono-culture economy (oil-driven) Majority of mental health financing goes to mental hospitals Proportion of government budget for health sector still below the Abuja declaration target of 15% Households are the main sources of funding for care of persons with mental disorders (OPP) Increasing recurrent expenditures compared to capital expenditures
*Political factors*	*Political factors*
The adoption of the revised mental health policy by National Council of Health in 2013 Increasing recognition and political support for Psycho-social interventions in the rebuilding of the north-east region Establishment of National Desk Office for Mental Health and, increasingly, at regional levels too Formation of the National Action Committee on Mental Health; which is a ministerial expert advisory body on mental health matters	Endemic corruption Bureaucratic bottlenecks and the slow pace of getting things done

**Table 2:** Opportunities and threats within the Nigerian Context

Opportunities	Threats
*Health system factors*	*Health system factors*
A new mental health bill is before the National Assembly Implementation of task-shifting and integration of mental health into primary care with training of primary health care providers on mental health using the mental health gap action programme intervention guide (mhGAP-IG) is increasingly becoming acceptable across the country The adoption of the mhGAP Implementation Plan by the National Council of Health in 2013 for the scaling up of mental health service in the country Implementation of health insurance schemes; and pilot studies of VCHIS have demonstrated promising results, which can improve access to MH services Projects funded by EU and other donor agencies are providing new evidence and models for scaling up mental health care services in Nigeria	Health insurance for the informal sector workers is yet to begin Spill-over effects of internal crises (insurgency) on the health sector with resultant displacement of larger populations
*Economic factors*	*Economic factors*
High projected economic growth Tax revenue expected to improve Massive investment in the power sector to boost generation capacity and productivity	Fall in international oil price Budget deficit and negative current account balance High levels of poverty, unemployment, adult illiteracy, and population growth Economic Recession in Nigeria from second quarter of 2016
*Political factors*	*Political factors*
Encouraging policy environment for health New UN resolution on NCDs, including the SDGs which include specific targets for mental health New health sector policies/plans focus on strengthening primary care and sustainable health financing Determination to fight corruption by the government	Insurgency and political tension and power struggles Low scores on polity, stability, and corruption indexes; instability of neighbouring countries Neglect of Mental Health in the 2014 National Health Act and the delay in passing the National Mental Health Bill by the National Assembly

Thus, the pertinent issues, as inferred from the SWOT analysis can be summarized under the following:

#### Governance and leadership

Mental health is gaining increasing policy attention. Furthermore, there has been support from various international donor agencies for research into mental health systems strengthening as well as the integration of mental health into primary care. The National Health Act of 2014 stipulates that 1% of the consolidated federal revenue will be set aside for primary care service (0.5% each) and the NHIS. This should ultimately translate into improved coverage for mental health which is the 9th component of primary care and is also covered under the NHIS. Implementation has been slow but positive across the country, with many states funding the establishment of: (a) State Primary Health Care Boards; and (b) State Health Insurance Schemes.

#### Financing: generation

Given the fact that Nigeria recently came out of economic recession, there may be minimal fiscal space for increased investment in health care and specifically for mental health. In addition, Nigeria‘s gradual economic recovery may not improve significantly in the immediate future, and continuous double-digit inflation rates may engender more fiscal and monetary control that may not favour the health sector.

In general, government spending on the direct needs of the population (% of GDP) and government spending on health (per capita) is currently low compared to other countries of the region. With the relatively low levels of current spending, there may technically be fiscal space for financing scaled-up mental health service delivery. This will require greater fiscal discipline and elimination of wastage. The government is also actively working to expand the tax coverage and this is already yielding increased revenue for the government. Special taxes such as corporate bodies’ tax and taxation on products injurious to health (such as tobacco and alcohol) may also be useful means of generating funds for investment in mental health care services specifically, and health care generally.

#### Financing: pooling

In appreciation of the fact that a high proportion of Nigerians live below the poverty line, and that out of pocket payments impoverish households, it is compelling to consider some form of social insurance scheme as a viable alternative for mental health care financing in Nigeria and other low- and middle-income countries.

The national health insurance scheme (NHIS) in Nigeria was enacted in 1999 [27], and is comprised of compulsory and voluntary contributions for different groups of participants. The groups are covered by three programme streams: Formal Sector Programme (FSP), Informal Sector Programme (ISP) and vulnerable groups programme (VGP). The FSP is compulsory for formal, public and private sector workers, and comprise two types of programmes: social health insurance (SHI) and private health insurance (PHI). While enrolment in SHI is mandatory for public sector workers, private sector workers are given the option of SHI or PHI enrolment. The ISPs are of two types vis: work–based health insurance (WBHI); and Community-based Health Insurance (CBHI). Membership of WBHI consists of individuals with common economic interests, residing in rural or urban areas; while membership of CBHI comprises people in the same location who enrol in a Mutual Health Association that can be registered at the local government area (LGA) and is required to have at least 500 members. The vulnerable group health insurance (VGHI) covers the permanently physically challenged, the aged, prisoners, and children under 5 years as well as pregnant women who are not covered by other schemes. It does not include persons with mental illness.

Contributions to NHIS are earnings–related. The employer pays 10% while the employee pays 5% representing 15% of the employee’s basic salary. The employer may decide to pay the entire contribution. The number of participants in NHIS has increased from less than 20 at the inception in 2006 to more than two million participants as at June, 2013. A total of 272, 068 civil servants (principal and dependants) were registered under the scheme in 2007. The number of enrollees from 2005 to 2007 was around 1,881,426 [27] and increased to 2,349,363 as at June 2013; given a growth rate of 24.9% from 2007 to 2013. Using, 2006 census figures [28], this is about 1.7% of the total population in Nigeria, as at 2013. The 2018 report on the functioning of Health Management Organizations (HMO) in Nigeria reports that 94.9% of Nigerians (approximately 170 million people) are not currently covered by any form of health insurance; and that 83% (149 million Nigerians) pay for their health care out of pocket [29]. This low insurance penetrance tempers enthusiasm about sustainable financing using the health insurance model. But the counterbalance for optimism is the recent trend towards encouraging social and community-based insurance schemes at district and state levels. It is also pertinent to note that mental health conditions are covered at all tiers of health care services (primary, secondary and tertiary) for specifically listed conditions such as schizophrenia, depression, bipolar disorder, organic psychiatric disorders, childhood disorders, and an umbrella term for ‘other specialized mental illnesses not already listed’.

Thus, the thrust of Governmental policy for the expansion of social health insurance (SHI) and establishment of voluntary contributory social health insurance together with community-based health insurance (CBHI) provides a promising mechanism for scaling-up investment in mental health and reducing out of pocket payments that may potentially impoverish households. A study indicated that efficient moral hazard (additional health care available to persons due to a fall in price of health care services following the purchase of health insurance) was high, especially with the recent expansion of health insurance in Nigeria [30]. As a result, health insurance can be a means of effective and sustainable financing for health care services. In order to achieve this objective and to reduce the burden of mental health financing, health insurance premiums can be subsidised to encourage coverage under the social health insurance and private health insurance. De-emphasizing cost sharing in the form of co-insurance which forms part of social health insurance policy in Nigeria may also suggest a promising future for mental health financing in Nigeria.

#### Provision of health coverage

Relevant indicators show that overall health coverage is weak and inequitable in Nigeria, with more inequities between the rich and the poor; as well as between rural and urban areas. A country with such weak and inequitable coverage of services faces real challenges in improving coverage for services in mental health. Indeed, access to mental health workers is low in comparison to other African and low-income countries. However, the successful integration of mental health care into primary care using the principle of task-sharing, an approach which has been successfully piloted in the country, may be a pragmatic means of improving access to mental health care services [31].

Furthermore, considering the high proportion of the populace living below the poverty line; and OOP as the most prevalent modality for purchasing health care, financial barriers to improved health coverage remains a source of concern. This can be potentially mitigated, via the expansion and effective, nationwide implementation of regional (state) health insurance schemes.

### Sustainable mental health financing in Nigeria: the way forward

This section reports on the outcome of interviews conducted using a Mental Health Financing Diagnostic Tool, aimed at gaining a deeper understanding of the process for health financing reform in general, but with specific focus on mental health financing in Nigeria. A total of 12 respondents (4 females and 8 male respondents) were interviewed, with the breakdown of respondents as follows: (a) 3 State Actors from the Ministry of Health—2 national and 1 regional; 3 State Actors from the Ministry of Finance—2 national and 1 regional (Director of a research institute); 5 Non-State Actors—3 national and 2 regional; and 1 Non-Health, Non-Finance State Actors—national. The Non-State Actors were drawn from 3 NGOs, as well as the WHO and World Bank Country Offices. The findings here are presented under 3 themes of perceived constraints, feasible recommendations for the attainment of increased and sustainable public and mental health financing as well as key elements for success.

- Perceived constraints to increased public and mental health financing.
  The low priority for mental health, was identified as major constraint as it directly impacts on resource allocation during budgetary planning. Thus, only about 3.3% of the health budget is allocated to mental health; out of which 90% goes to support the specialist neuropsychiatric hospitals for payment of salaries and other recurrent expenditure [19]. This is explained by one of the respondents below:

*“Mental health is just a very small aspect, when compared to the entire portfolio that we handle. The focus is more on infectious diseases such as Tuberculosis, Malaria, HIV/AIDS and so on.”*—***Non State Actor 1 (female).***

Second, priorities are frequently donor-driven, rather than determined on the strength of available research evidence. Lastly, fiscal pressures due to the economic recession and fluctuations in the international prices of oil has resulted in reduced government revenue and this was also identified as a constraint to increasing government funding for public, as well as mental health.

b. Feasible opportunities and recommended strategies for sustainable increase in public health as well as mental health financing. Several opportunities and recommendations were generated. First, the draft national strategic health development plan (NSHDP) for 2017–2022 included mental health for the first time. This should translate into the assurance of a dedicated funding envelope for mental health services. Second, improved political will to designate mental health as an area of government priority, will create the opportunity to attract counterpart funding from donor agencies—including loans from the World Bank. This advocacy can be championed by technocrats, service user groups and professional associations, to create more awareness for mental health; with the legislators and policy makers as the target audience. An opportunity for funding support from the World Bank is explained thus:

*“The World Bank functions by providing loans and technical assistance for the attainment of health goals as stipulated by the government of the given country. So, if the government has not approached the World Bank to provide loans or funds to support mental health activities in the country, it will not happen. But, there is an opportunity if the government applies for it.”*—***Non State Actor 5 (male).***

Third, there is an opportunity to link mental health with other health conditions that are currently being funded; and then outlining the salient mental health aspects that should be taken into consideration and implemented. For example, perinatal depression could be tied to maternal and child health budgets; or depression linked with chronic diseases, such as within the context of HIV/AIDS funding. This was expressed by a respondent as quoted here:

*“The key is to link mental health with other related health conditions that are currently being funded and then specify that the mental health components should be implemented. Such as linking maternal depression to maternal and child health budgets; or depression in chronic diseases, within the context of HIV/AIDS funding. But if we are waiting to have special funds only for mental health, it will not be a realistic expectation…at least for now”*—***Non State Actor 4 (female).***

Fourth, integrating mental health into primary care, is a strategy that can potentially result in an indirect increase in public health funding for mental health. This is because the newly ratified National Health Act of Nigeria provides for the allocation of 1% of the Consolidated Federal Revenue with 0.5% for primary care and 0.5% for NHIS. Mental health is already the 9th component of primary care, but this is currently not being implemented. If primary care is better funded and mental health is effectively implemented within the primary care system, services will naturally be scaled up, due to the increased resources available for primary health care. Ultimately, this will result in a reduction of the treatment gap for mental disorders.

Fifth, improved taxation such as on every mobile phone airtime recharge, and ‘sin tax’ such as a token on alcoholic drinks spread across the country’s huge population can potentially generate huge resources that may be deployed for improved public health financing—including mental health. This suggestion is explained by a respondent below:

*“Introduction of new tax such as telecommunications tax (on mobile phone recharge) and ‘Sin Tax’ such as 1 naira on every bottle of beer can be a painless means of pooling huge resources that can be deployed for improved financing of public health generally, and mental health specifically.”*—***State Actor Finance 1 (male)***

Sixth, plans are underway to create a Division for Mental Health within the Federal Ministry of Health, unlike the current situation where a Desk Officer exists under the non-communicable diseases section of the Directorate of Public Health. If this materializes, it will raise the profile of mental health, and the Division will have a dedicated budget line and funding for its activities. This should enhance the strengthening of mental health systems in Nigeria.

Lastly, the expansion of health insurance coverage to include the informal sector and the huge proportion of uninsured citizens will increase the pool of funds channelled to funding public health and mental health. Given the greater concentration of the populace in the informal sector and the high level of poverty in Nigeria, there is an absence of a robust tax base for financing health services through taxation alone. More so, reliance on donor funding is neither sustainable nor reliable, due to vulnerability to external shocks such as withdrawal or cancellation of funding. Thus, an improved coverage via social health insurance is an important strategy to raise the pool of available resources for financing health services—including mental health.

c. Key considerations for successful implementation of sustainable mental health financing.
  Pragmatic considerations that were suggested by the respondents are presented here. First, sustained advocacy with involvement of opinion leaders such as religious and community leaders (the Sultan [head] of the Muslims in the country; the head of the Christian Association of Nigeria etc.), and celebrities (Musicians, Actors etc.) in order to counter stigma against mental health and promote funding and improved services—similar to what was has been successfully achieved for HIV/AIDS.
  Second, ensure a specified budget line for mental health (either in the NSHDP or as a Division in the FMOH) that will guarantee sustainable financing for mental health services and programmes. Third, synergy and collaboration between the Government (Health and other relevant Ministries) and stakeholders such as international donor agencies, the NHIS, the media, the legislative health committees (national and regional levels) and the National Primary Health Care Development Agency (NPHCDA) is crucial to ensure a multiplier effect on current efforts. Fourth, efficiency and transparency in the utilization of allocated resources must be consistently demonstrated. This will encourage the attraction of both domestic and foreign funding for mental health services. World Bank loans for example, are closely monitored and tracked to ensure fidelity and transparency. This is illustrated by a respondent quoted below:

*“As far as I know, there is minimal to non*-*existent budget tracking and performance monitoring. This needs to be implemented, along with plugging massive leakage and inefficiencies in the system to ensure good value for money on investments.”*—***State Actor Finance 3 (male).***

## Discussion

The Emerald project aimed to strengthen mental health systems by addressing the various components necessary to actualize a reduction in the burden of mental disorders in low and middle income countries including Nigeria, through the scaling up of mental health services. One of the Emerald Work Packages (WP3), focused on several aspects of generating resources for mental health services in an equitable, fair and sustainable manner. This section touches briefly on the previous outputs of WP3 to provide background context, and then presents a discussion of the recommended strategies for sustainable mental health financing in Nigeria.

Task 1 of WP 3 generated results about the price tag of scaling up mental health services and the expected health gains using the OneHealth Tool. Task 2 reports on the economic consequences of mental illness (depression) on households; while task 3 explores the macro-economic and financing considerations of sustainable financing to support mental health services scale up in Nigeria.

Results from WP3.1 revealed that substantial financial resources are required for scaling-up mental health services in Nigeria. The estimated total cost of scaling up interventions for psychosis, depression and epilepsy (including intervention and programme costs) is about N27.8b ($78.9M). This translates to about N45.5 ($0.14) per capita. With the 2017 Nigeria’s budgetary allocation to public health as N304B ($0.856m at N355 per $1), the total cost is about 10% of the government’s 2017 public health budget. The average healthy years gained for the whole population is about 30, 318.0 years for psychosis, about 379, 364.0 years for depression and about 9,224.0 years for epilepsy. Committing about 10% of total public health budget to mental health, compared to the current level of 3.3%, is therefore justifiable, given the average healthy years gained as exemplified by just three conditions cited here [13].

The economic consequences of mental health on households of persons with depression (as an index condition of study) compared to households of persons without depression (WP3.2 output) presents evidence of a negative economic impact of the mental disorder. For instance, over half (55%) of the households with depression are living below poverty line, compared to less than a third (31%) of households of persons without depression. Other details will be presented in a forthcoming paper.

**Fig. 4:**
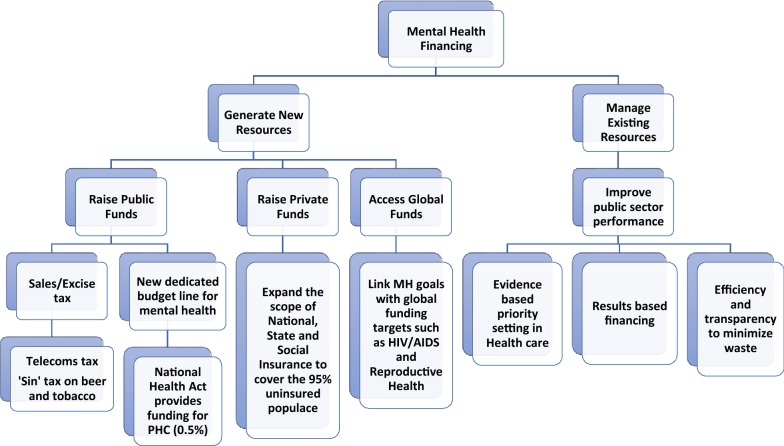
Proposed model of mental health financing for Nigeria

WP3.3 outcomes presented the background context for the country via the situational analysis report as well as the SWOT analysis. Even more importantly, the results of the interviews with selected experts identified the most feasible options for sustainable mental health financing in Nigeria, which can be categorised into domestic financing, bilateral/multilateral funding, and other more innovative forms of financing (see Fig. 4). A limitation of the situational analysis is the restriction to available official reports and estimates, some of which are not very recent. Even though, it was reasonably assumed that the situation has remained fairly consistent over time, more recent reports would have been more accurate.

## Proposed strategies for sustainable mental health financing in Nigeria

Inadequate means of paying for health care services has subjected many households in low and middle income countries such as Nigeria to financial difficulties and pushed them into poverty. Insufficient human and material resources and the associated limited coverage for mental, neurological and substance use (MNS) disorders have resulted in adverse economic consequences for persons affected by mental disorders and their households. The Emerald project evaluated the conceptual framework for achieving equitable and sustainable mental health financing, by performing a situational analysis of the key dimensions of UHC, financial risk protection and access to services within the broader health system characteristics, constraints, determinants and capacities (including the macroeconomic and fiscal environment) of each participating country. It also explored potential strategies for mental health financing and increased financial protection via in-depth interviews with key stakeholders.

The most salient inference from the findings in Nigeria, is that public health insurance remains the most feasible and sustainable financing intervention for scaling up mental health care services. It is the only health financing form that possesses the important characteristics of universality, price regulation, open enrolment, and a defined and regulated benefits package expected of any dynamic health financing means. Therefore, an important suggestion here is that health insurance should be extended to accommodate both formal and informal sector employees with little concern for excess utilization of health care services due to the availability of health insurance. The expansion may be achieved by subsidising the insurance premium under social (public) health insurance and to a limited extent, private health insurance. The NHIS scheme in Nigeria currently covers only about 5% of the country’s population. Social health insurance systems have four common features: universality (compulsory insurance with subsidization of the sick by the healthy), price regulation (to ensure risk solidarity usually combined with some form of risk compensation for insurers with relatively many high risks insured), open enrolment, and a defined and regulated benefits package. The recommendation of health insurance is supported by a pilot study of VCHIS [32] which shows a high degree of readiness for participation in health insurance scheme. The revenue-raising potential of health insurance is high compared to other forms of financing and is also capable of providing more financial protection and coverage for persons with mental disorders. It promotes equity and offers other gains as well, such as human rights protection, poverty reduction, reduced OOPs, and can meet the needs of vulnerable groups. An expanded insurance coverage will substantially assist about 55.2% of the households with depression cases who currently live below the poverty line—based on the findings from our baseline household survey in Task 3.2.

It is also important to engage in advocacy targeted at stakeholders (including policymakers and the general public) in the country, to improve their awareness of the burden of mental health care challenges in Nigeria. The advocacy should seek to reduce the ignorance and stigma attached to mental illness and generate more commitment from government at all levels for mental health care. Stakeholders should appreciate the costs (both explicit and implicit costs in terms of loss of income, productivity, life expectancy etc.) and benefits (in terms of increased life expectancy and increased societal welfare) of providing services for mental disorders. This readily translates into improved productivity with a positive economic impact at all levels—from households, to local government, state government and the national government. It may further require lobbying members of the national assembly (parliament) to pass a legislation giving mental health a special status both in context and content to ensure more commitment to mental health financially and otherwise. A good starting point would be ensuring the passage of the revised Mental Health Bill.

While expanding health insurance is agreed as the best option for sustainable financing for mental health care services, other suggestions and recommendations are also very feasible and pragmatic, and these are presented below.

## Recommendations

### Political strategies

First, increase advocacy and lobbying for mental health using respected public figures such as religious leaders. A technical group or MH alliance should be established to drive this agenda forward. This role can be taken up by the National Mental Health Action Committee, which already brings diverse stakeholders together. Second, concerted lobby for the establishment of a sin tax and telecommunications tax, as means of raising public funds for investment in health care generally, and mental health specifically should be pursued. A healthy nation is a productive one, and health care is a fundamental human right that should be of concern to political leaders.

### Health system strategies

First, advocate for the establishment of a Mental Health Division in the FMOH and lobby for a dedicated mental health budget as a health system governance requirement. Second, aim for the integration of mental health into globally funded and on-going programs such as HIV/AIDS and Maternal and Child Health programs. Third, ensure effective implementation of mental health as the 9th component of primary care in Nigeria; via the newly established State Primary Health Care Development Agencies. This will ensure that mental health becomes eligible for the 0.5% funding to primary health care that is assured in the National Health Act of 2014. Fourth, ensure the development of a mental health plan to accompany the revised mental health policy of 2013. The plan should include actionable financing streams for mental health. Fifth, the revised mental health legislation is fundamental to guaranteeing effective mental health services that promotes dignity and adopts a rights-based approach. All efforts to ensure its speedy passage should be deployed. Sixth, multi-sectoral collaboration, both within the health sector and outside the health sector (with the media, legislative arm, NGOs, religious bodies), will be useful to generate consensus and synergy. Seventh, efficiency in the utilization of allocated resources needs to be consistently and transparently demonstrated. Waste or mismanagement of health care funds should be eliminated; and results-based financing encouraged. Successful integration projects may be demonstrated with pilot mhGAP intervention projects across the country; and presented as proof of concept results to encourage further investments. Eight, adopt and learn from the highly effective and rapid health system response to infectious disease outbreaks such as Ebola, and borrow principles for application to mental health prevention and promotion activities. Lastly, advocate for the routine deployment of evidence-based, priority setting in the health care sector, for instance, using the compelling results from Task 3.1 and 3.2 to make a case for improved mental health financing.

### Financing strategies

A potential strategy is to solicit for donor funding from agencies such as the World Bank, for psycho-social rehabilitation in the north east, as a means of generating revenue. The funds can then be applied towards ‘building back better’, the mental health systems in the most affected region, as well as across the country. Another viable strategy would be the expansion of health insurance schemes for wider coverage of the population. This would ensure a reduction in the proportion missing out of qualitative health care—including mental health care, due to financial constraints and the current system of OOP. Lastly, the introduction of telecommunication tax; and ‘sin tax’ on alcohol and tobacco, may be relatively painless means of improving government revenue for investment in public health—including mental health.

## Conclusion

There are several constraints to increasing financing for mental health services scale up in Nigeria. But several opportunities and innovative strategies can also be deployed, despite the challenges. These strategies derive from a consultative process with relevant experts, and they represent feasible and actionable measures that can be implemented to increase mental health service financing, reduce health-related financial burden on households, increase help-seeking and access to quality mental health care and, ultimately, reduce the large treatment and financing gap for mental disorders in Nigeria.

## Data Availability

The datasets generated and analysed from the key informant interviews are not publicly available due to the presence of information that could compromise research participant privacy but could be made available from the corresponding author on reasonable request, and with consent from participants. The secondary data generated and analysed from online and printed data, grey literature, government documents and policies as well as online databases are already in the public domain as specified in the reference list.

## Abbreviations

CBHI: community-based health insurance
Emerald: emerging mental health systems in low and middle income countries
FMOH: Federal Ministry of Health
FSP: formal sector programme
GDP: gross domestic product
HDI: Human Development Index
HMO: Health Management Organization
ISP: informal sector programme
KII: key informant interviews
LGA: local government area
LMIC: low and middle income countries
MNS: mental, neurological and substance use
NHIS: national health insurance scheme
NSHDP: national strategic health development plan
OOP: out of-pocket payment
PHI: private health insurance
SDG: sustainable development goals
SHI: social health insurance
SWOT: strengths, weaknesses, opportunities and threats
UHC: universal health coverage
VGHI: vulnerable group health insurance
VGP: vulnerable groups programme
WBHI: work–based health insurance
WHO: World Health Organization
